# Inflammatory Effects of Menthol vs. Non-menthol Cigarette Smoke Extract on Human Lung Epithelial Cells: A Double-Hit on TRPM8 by Reactive Oxygen Species and Menthol

**DOI:** 10.3389/fphys.2017.00263

**Published:** 2017-04-27

**Authors:** An-Hsuan Lin, Meng-Han Liu, Hsin-Kuo B. Ko, Diahn-Warng Perng, Tzong-Shyuan Lee, Yu Ru Kou

**Affiliations:** ^1^Department of Physiology, School of Medicine, National Yang-Ming UniversityTaipei, Taiwan; ^2^Department of Chest Medicine, Taipei Veterans General HospitalTaipei, Taiwan

**Keywords:** cigarette smoke, menthol, TRPM8, reactive oxygen species, calcium, signaling pathway, lung inflammation, lung epithelial cell

## Abstract

Clinical studies suggest that smokers with chronic obstructive pulmonary disease who use menthol cigarettes may display more severe lung inflammation than those who smoke non-menthol cigarette. However, the mechanisms for this difference remain unclear. Menthol is a ligand of transient receptor potential melastatin-8 (TRPM8), a Ca^2+^-permeant channel sensitive to reactive oxygen species (ROS). We previously reported that exposure of human bronchial epithelial cells (HBECs) to non-menthol cigarette smoke extract (Non-M-CSE) triggers a cascade of inflammatory signaling leading to IL-8 induction. In this study, we used this *in vitro* model to compare the inflammatory effects of menthol cigarette smoke extract (M-CSE) and Non-M-CSE and delineate the mechanisms underlying the differences in their impacts. Compared with Non-M-CSE, M-CSE initially increased a similar level of extracellular ROS, suggesting the equivalent oxidant potency. However, M-CSE subsequently produced more remarkable elevations in intracellular Ca^2+^, activation of the mitogen-activated protein kinases (MAPKs)/nuclear factor-κB (NF-κB) signaling, and IL-8 induction. The extracellular ROS responses to both CSE types were totally inhibited by N-acetyl-cysteine (NAC; a ROS scavenger). The intracellular Ca^2+^ responses to both CSE types were also totally prevented by NAC, AMTB (a TRPM8 antagonist), or EGTA (an extracellular Ca^2+^ chelator). The activation of the MAPK/NF-κB signaling and induction of IL-8 to both CSE types were suppressed to similar levels by NAC, AMTB, or EGTA. These results suggest that, in addition to ROS generated by both CSE types, the menthol in M-CSE may act as another stimulus to further activate TRPM8 and induce the observed responses. We also found that menthol combined with Non-M-CSE induced greater responses of intracellular Ca^2+^ and IL-8 compared with Non-M-CSE alone. Moreover, we confirmed the essential role of TRPM8 in these responses to Non-M-CSE or M-CSE and the difference in these responses between the both CSE types using HBECs with TRPM8 knockdown and TRPM8 knockout, and using HEK293 cells transfected with hTRPM8. Thus, compared with exposure to Non-M-CSE, exposure to M-CSE induced greater TRPM8-mediated inflammatory responses in HBECs. These augmented effects may be due to a double-hit on lung epithelial TRPM8 by ROS generated from CSE and the menthol in M-CSE.

## Introduction

Chronic obstructive pulmonary disease (COPD) is characterized by persistent lung inflammation, and cigarette smoking is its major etiologic factor (Chung and Adcock, 2008). The rate of menthol cigarette smoking has increased in recent years in the US (Caraballo and Asman, 2011). Smokers have been consistently cautioned regarding the more harmful impact of menthol cigarette smoking than non-menthol cigarette smoking (Hoffman, 2011; Besaratinia and Tommasi, 2015). A recent large cohort study (Park et al., 2015) have reported that menthol cigarette smokers presented more frequent severe exacerbations of COPD during longitudinal follow-up compared with non-menthol cigarette smokers. This finding suggests that menthol cigarette smoking in the COPD population may result in more severe lung inflammation. Inflammation of lungs with COPD is regulated by a complex mechanism involving different cells and inflammatory mediators (Chung and Adcock, 2008). For example, upon direct stimulation by cigarette smoke, the chemokine interleukin-8 (IL-8) released from lung epithelial cells plays a vital role in regulating lung inflammation (Mossman et al., 2006; Tang et al., 2011; Liu et al., 2014; Wu et al., 2014; Lin et al., 2015). Induction of chemokines and cytokines in lung epithelial cells by cigarette smoke is mainly regulated by redox-sensitive signaling pathways (Mossman et al., 2006). This phenomenon is due to the fact that cigarette smoke is a potent oxidant that can increase both extracellular and intracellular reactive oxygen species (ROS) (Nakayama et al., 1989; Wu et al., 2014; Lin et al., 2015). Increased ROS in the lung epithelial cells may activate ROS-sensitive signaling pathways, such as mitogen-activated protein kinases (MAPKs), and downstream transcriptional factors, such as nuclear factor-κB (NF-κB) (Mossman et al., 2006). These events then ultimately upregulate inflammatory gene expression (Mossman et al., 2006; Tang et al., 2011; Liu et al., 2014; Wu et al., 2014; Lin et al., 2015). Although these pathogenetic mechanisms have been relatively elucidated, the negative impact of menthol compared with those of non-menthol cigarette smoke on lung inflammation remains unknown.

Menthol is a ligand of transient receptor potential melastatin-8 (TRPM8), a Ca^2+^-permeant, non-selective cation channel mainly expressed in primary sensory neurons (Journigan and Zaveri, 2013). Neuronal TRPM8 is also known as the cold receptor, and its activation leads to an increase in intracellular Ca^2+^ via influx, which in turn results in generation of neural impulses in sensory fibers (Journigan and Zaveri, 2013). Recent studies have demonstrated that TRPM8 is also expressed in various non-neuronal cells (Journigan and Zaveri, 2013), including lung epithelial cells (Sabnis et al., 2008a,b; Li et al., 2011, 2014), airway smooth muscle cells (Zhang et al., 2016), and pulmonary vascular smooth muscle cells (Yang et al., 2006; Johnson et al., 2009). Activation of the TRPM8 in the lung epithelial cells by cold temperature or menthol leads to the upregulation of the expression of cytokine and chemokine genes, including IL-8 (Sabnis et al., 2008a,b), and mucus hypersecretion (Li et al., 2011). All these cold- or menthol-induced consequences can be inhibited by antagonists or small interfering RNA (siRNA) targeting TRPM8 (Sabnis et al., 2008a,b; Li et al., 2011), suggesting the inflammatory role of lung epithelial TRPM8. Additionally, exposure to H_2_O_2_, a type of ROS, can activate TRPM8 in the uroepithelium (Nocchi et al., 2014). These observations promote the novel hypothesis that, in addition to ROS generated by cigarette smoke, the menthol in the smoke may act as another stimulus to induce inflammation in lung epithelial cells. However, this hypothesis remains to be proven.

In this study, we hypothesized that menthol cigarette smoke could induce more severe inflammation in lung epithelial cells than non-menthol cigarette smoke via a more vigorous activation of inflammatory signaling. This phenomenon was inferred to be due to a double-hit on lung epithelial TRPM8 by ROS and menthol. To test this hypothesis, we used a well-established *in vitro* model involving primary human bronchial epithelial cells (HBECs) (Tang et al., 2011; Liu et al., 2014; Wu et al., 2014; Lin et al., 2015). We exposed these cells to menthol cigarette smoke extract (M-CSE) or non-menthol cigarette smoke extract (Non-M-CSE). Responses of extracellular ROS, intracellular ROS, intracellular Ca^2+^, MAPKs/NF-κB signaling, and IL-8 to these CSEs were compared. Various interventions were used to delineate the role of ROS, menthol, and TRPM8 in the more negative impact of M-CSE.

## Methods

### Reagents

Antibodies (Abs) and ELISA kits for detecting IL-8 and AMTB hydrochloride (AMTB) were purchased from R&D Systems (Minneapolis, MN, USA). Rabbit Ab against c-Jun N-terminal kinases (JNK) was obtained from Cell Signaling (Beverly, MA, USA). Mouse Ab against phospho-JNK was purchased from BD Biosciences (San Jose, CA, USA). Mouse Abs against extracellular signal-regulated kinase (ERK), phospho-ERK, histone 1 (H-1), and rabbit Ab against p65 and nuclear factor-like 2 (Nrf2) were obtained from Santa Cruz Biotechnology (Santa Cruz, CA, USA). Mouse Ab against α-tubulin, mouse Ab against FLAG, ethylene glycol tetraacetic acid (EGTA), N-acetyl-cysteine (NAC), apocynin, and L-menthol (purity > 99%, FCC) were purchased from Sigma-Aldrich (St. Louis, MO, USA). Rabbit Ab against TRPM8 and 4-Hydroxynonenal (4-HNE) were obtained from Abcam (Cambridge, MA, USA). The Screen Quest™ Fluo-8 Medium Removal Calcium Assay Kit was purchased from AAT Bioquest (Sunnyvale, CA, USA). The membrane-permeable probe hydroethidine (HE) was purchased from Molecular Probes (Eugene, OR, USA). Scramble and TRPM8 siRNAs were obtained from GE Dharmacon (Lafayette, CO, USA). INTERFERin siRNA transfection reagent was purchased from Polyplus (New York, NY, USA).

### Preparation of Non-M-CSE or M-CSE

Non-M-CSE or M-CSE was freshly prepared on the day of the experiment as previously described (Tang et al., 2011; Wu et al., 2014). Smoke (1,000 ml) generated from two burning cigarettes without filters were sucked at a constant flow rate (8 ml/s) into a syringe and then bubbled into a tube containing 20 ml of serum-free medium. One brand of non-menthol cigarette (West Rich Blue) and one brand of menthol cigarettes (Marlboro Black Menthol) were used, because these two brands have the same contents of tar (8 mg) and nicotine (0.6 mg), lengths without filter (5.6 cm), and diameters (0.8 cm) in each cigarette. These brands also have very similar amounts of tobacco (~600 mg) in each cigarette. Non-M-CSE or M-CSE solution was sterilized using a 0.22-μm filter (Millipore, Bedford, MA), and the pH of the solution was adjusted to 7.4. The optical density of the Non-M-CSE or M-CSE solution was determined by measuring the absorbance at 302 (Non-M-CSE vs. M-CSE, 3.24 ± 0.01 vs. 3.23 ± 0.01, *n* = 5) (Yamaguchi et al., 2007) or 320 nm (Non-M-CSE vs. M-CSE, 3.34 ± 0.01 vs. 3.32 ± 0.01, *n* = 5) (Facchinetti et al., 2007). Little difference was found between the two preparations. The Non-M-CSE or M-CSE solution was considered 100% Non-M-CSE or M-CSE and further diluted with serum-free medium to desired concentrations. The diluted solutions were then used to treat HBECs for different durations.

### Cell culture

HBECs (Cascade Biologics, Portland, OR, USA) were cultured in epithelial cell growth medium (medium 200; Cascade Biologics, USA) at 37°C in an incubator with 5% CO_2_. The medium contained 10% fetal bovine serum (FBS), 1X low serum growth supplement, 100 U/ml penicillin, 100 mg/ml streptomycin, and 0.25 mg/ml amphotericin B (Biological Industries, Kibbutz Beit Haemek, Israel). Human embryonic kidney 293 cells (HEK293, ATCC® CRL-1573™) were cultured in Dulbecco's modified Eagle's medium (Cascade Biologics, USA) at 37°C in an incubator with 5% CO_2_. The medium was supplemented with 10% FBS, 100 U/ml penicillin, 100 mg/ml streptomycin, and 0.25 mg/ml amphotericin B.

### siRNA transfection in HBECs

HBECs were transfected with either siGENOME SMARTpool human TRPM8 siRNA or non-targeting SMARTpool control siRNA using INTERFERin siRNA transfection reagent for 24 h. The SMARTpool human TRPM8 siRNA consisted of D-006517-01, D-006517-02, D-006517-03, and D-006517-04. The nucleotide target sequence of D-006517-01 was GGAAUCAGCUAGAGAAGUA, while that of D-006517-02 was CGAAUGUUCUCACCUAUUA. The nucleotide target sequence of D-006517-03 was GAAGAAACCUGUCGACAAG, while that of D-006517-04 was GCAAUGGUAUGGAGAGAUU.

### CRISPR/Cas9-mediated TRPM8 knockout in HBECs

For CRISPR-Cas9-mediated gene knockout (Chu et al., 2015), control and TRPM8 Double Nickase plasmid were purchased from Santa Cruz Biotechnology (sc-437281 and sc-401744-NIC; Santa Cruz) and transfected in HBECs using *Trans*IT-X2® Dynamic Delivery System (Mirus; Madison, WI, USA) for 48 h according to the manufacturer's manual. Briefly, 2 × 10^5^ HBECs were plated onto 6 × 30-mm well plates and allowed to grow to 80% confluence. *Trans*IT-X2 (7.5 μl) was added to 2.5 μg of control or TRPM8 Double Nickase plasmid. The complexes were incubated at room temperature for 20 min and then overlaid onto HBECs. The plates were then incubated at 37°C under 5% CO_2_ for 48 h. Stably transfected clones were selected by adding puromycin (0.75 μg/ml) for 4 weeks. The TRPM8 Double Nickase plasmid (sc-401744-NIC) contained a target-specific 20 nt guide RNA (gRNA) to form the Cas9/gRNA complex to disrupt the expression of human TRPM8 (hTRPM8) gene. The control Double Nickase plasmid (sc-437281) contained a non-targeting 20 nt scramble gRNA designed as a negative control. Thus, the Cas9/gRNA complex did not recognize any DNA sequence and would not bind or cleave genomic DNA.

### Transfection of hTRPM8 vector in HEK293 cells

HEK293 cells (1 × 10^6^) were transfected with 5 μg of empty vector (pCMV6) or hTRPM8 (C-terminal FLAG tag) vector obtained from OriGene Technologies (Rockville, MD, USA) or for 24 h using Lipofectamine 2000 (Invitrogen; Carlsbad, CA, USA) following the manufacturer's recommended procedure.

### Measurement of intracellular Ca^2+^ levels

Intracellular Ca^2+^ levels were determined using the Screen Quest™ Fluo-8 Medium Removal Calcium Assay Kit according to the manufacturer's instructions.

### Measurement of extracellular and intracellular ROS levels

The membrane-permeable probe HE was used to assess levels of ROS using a previously described method (Liu et al., 2005). HE is converted by ROS to red fluorescent ethidium (ETH) (Benov et al., 1998). For the *in vitro* study, HBECs were incubated in culture medium containing 10 μM HE at 37°C for 30 min. After stimulation with Non-M-CSE or M-CSE for the desired time, the culture medium was removed to measure the extracellular ROS levels. The cells were washed and detached with trypsin/EDTA to measure the intracellular ROS levels. Fluorescence intensities of the culture medium and cell samples were then analyzed using a multilabel counter (PerkinElmer, Waltham, MA, USA). Cell images were also obtained using a Nikon TE2000-U florescence microscope (Tokyo, Japan).

### Western blot analysis

Cell lysates were prepared using cell lysis buffer. Nuclear extracts were prepared using a previously reported method (Beg et al., 1993) with modifications. Aliquots of cell lysates or nuclear extracts were separated by 8–12% SDS-PAGE and then transblotted onto Immobilon™-P membrane (Millipore). After being blocked with 5% skim milk, the blots were incubated with various primary antibodies and then with appropriate secondary antibodies. The specific protein bands were detected using an enhanced chemiluminescence kit (PerkinElmer), followed by quantification using ImageQuant 5.2 software (GE Healthcare Bio-Sciences, Philadelphia, PA, USA).

### Reverse transcription-polymerase chain reaction (RT-PCR)

Total RNA was isolated from cells using Tri reagent and converted into cDNA using reverse transcriptase (Biolabs, Ipswich, New England) and oligo-dT as the primer. The resultant cDNAs were then used as templates for the semi-quantitative PCR. PCR was performed in a DNA Thermal Cycler (Biometra Tpersonal, Horsham, PA, USA) using the following program: 94°C for 5 min, followed by 35 cycles of 94°C for 15 s, 65°C for 30 s, 72°C for 30 s, and then a final single cycle of 72°C for 7 min. The nucleotide sequences of the primers were as follows: for TRPM8, sense, 5′-CCT GTT CCT CTT TGC GGT GTG GAT-3′ and anti-sense, 5′-TCC TCT GAG GTG TCG TTG GCT TT-3′; and for β-actin, sense, 5′-GAT CCT CAC CGA GCG CGG CTA CA-3′ and anti-sense, 5′-GCG GAT GTC CAC GTC ACA CTT CA-3′.

### Measurement of IL-8 concentration

The concentrations of IL-8 in the culture media were measured using an ELISA kit according to the manufacturer's instructions.

### Statistical analysis

The results are presented as means ± SEM. Statistical evaluations involved one-way ANOVA followed by Dunnett's test or Fisher's least significant difference procedure for multiple comparisons as appropriate. Differences were considered statistically significant at *p* < 0.05.

## Results

### Role of ROS and TRPM8 in the induction of IL-8 by M-CSE or Non-M-CSE in HBECs

**Figure 1:**
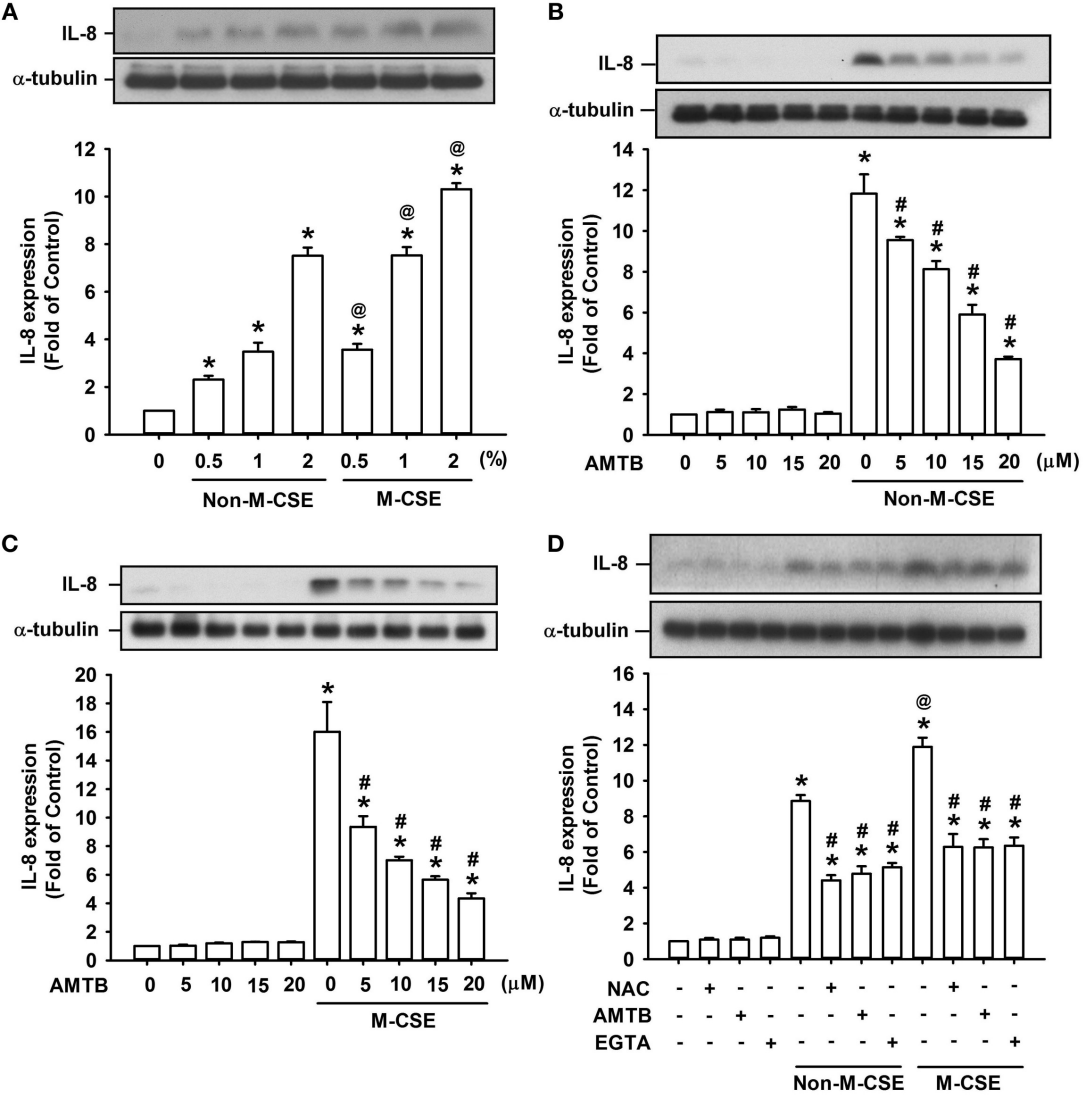
**Roles of reactive oxygen species (ROS) and TRPM8 in IL-8 induction by non-menthol cigarette smoke extract (Non-M-CSE) and menthol cigarette smoke extract (M-CSE) in human bronchial epithelial cells (HBECs). (A)** Cells were exposed to medium alone, 0.5–2% Non-M-CSE or M-CSE for 24 h. **(B,C)** Cells were pretreated with 5–20 μM AMTB (a TRPM8 antagonist) for 1 h and then exposed to medium alone, 1% Non-M-CSE or 1% M-CSE for 24 h. **(D)** Cells were pretreated with N-acetyl-cysteine (NAC, a ROS scavenger; 1 mM), AMTB (20 μM), or EGTA (an extracellular Ca^2+^ chelator; 500 μM) for 1 h and then exposed to medium alone, 1% Non-M-CSE, or M-CSE for 24 h. Protein levels of IL-8 were analyzed by Western blot. Data from each group are means ± SEM from four independent experiments. ^*^p < 0.05 vs. the medium group **(A–D)**; ^@^*p* < 0.05 vs. the Non-M-CSE group with the same concentrations **(A,D)**; #*p* < 0.05 vs. the same type of CSE group without pretreatment **(B–D)**.

Analyses of cell lysates showed that exposure of HBEC to various concentrations (0.5, 1, and 2%) of Non-M-CSE or M-CSE for 24 h increased the protein level of IL-8 in HBECs in a concentration-dependent manner (Figure 1A). Comparisons of the IL-8 responses to Non-M-CSE and M-CSE revealed that M-CSE exerted a greater effect at each concentration tested (Figure 1A). For example, the IL-8 responses to 1% Non-M-CSE and M-CSE were 360% and 735%, respectively, of the basal level. Moreover, the increased IL-8 production induced by 1% Non-M-CSE (Figure 1B) or 1% M-CSE (Figure 1C) was concentration-dependently attenuated by pretreatment with AMTB (5–20 μM), a specific TRPM8 antagonist (Lashinger et al., 2008). For example, the responses to 1% Non-M-CSE and 1% M-CSE were reduced to 354 and 353% of the basal level, respectively, by 20 μM AMTB. Thus, we used 1% CSE and 20 μM AMTB as the standard stimulus and treatment, respectively, for subsequent experiments. Considering that ROS may activate TRPM8 to promote the influx of Ca^2+^ (Nocchi et al., 2014), we further characterized the TRPM8-mediated induction of IL-8 by both CSE types. Further analysis revealed that increased IL-8 production induced by Non-M-CSE or M-CSE was significantly suppressed by scavenging ROS with NAC (Non-M-CSE vs. M-CSE, 401% vs. 571% of the basal level), antagonizing TRPM8 with AMTB (Non-M-CSE vs. M-CSE, 439% vs. 573% of the basal level), or removing extracellular Ca^2+^ with EGTA (Non-M-CSE vs. M-CSE, 432% vs. 534% of the basal level) to similar levels. No difference was detected in the residual responses to Non-M-CSE and M-CSE (Figure 1D). By contrast, pretreatment with NAC, AMTB, or EGTA did not affect the basal expression of IL-8 (Figure 1D).

### TRPM8-knockdown- or TRPM8-knockout-mediated suppression of the induction of IL-8 by M-CSE or Non-M-CSE in HBECs

**Figure 2:**
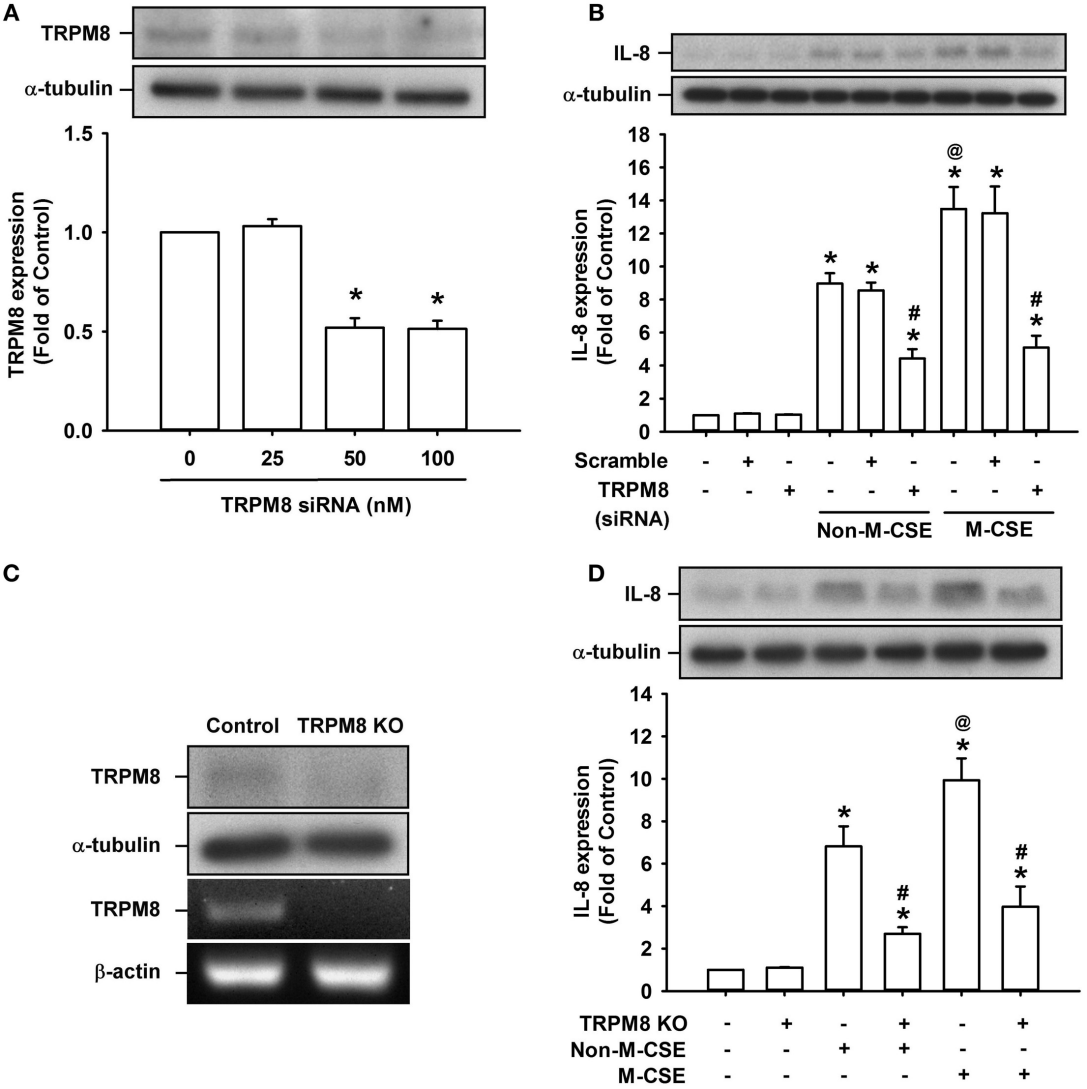
**Suppression of IL-8 induction by M-CSE or Non-M-CSE in HBECs caused by knockdown or knockout of TRPM8. (A)** Cells were incubated with or without TRPM8 siRNA for 24 h. **(B)** Cells were pretreated with 50 nM TRPM8 siRNA or scramble siRNA for 24 h and then exposed to medium alone, 1% Non-M-CSE or 1% M-CSE for 24 h. **(C)** Cells were transfected with control plasmid (control) or TRPM8 Double Nickase plasmid (KO). **(D)** Cells were transfected with control plasmid or TRPM8 KO for 48 h and then exposed to medium alone, 1% Non-M-CSE, or 1% M-CSE for 24 h. Protein levels of TRPM8 or IL-8 were analyzed by Western blot in all panels. mRNA levels of TRPM8 were analyzed by RT-PCR in **(C)**. Data from each group are means ± SEM from four independent experiments. ^*^p < 0.05 vs. the medium group **(A,B,D)**; ^@^*p* < 0.05 vs. the Non-M-CSE group without pretreatment **(B,D)**; #*p* < 0.05 vs. the same type of CSE group without pretreatment **(B,D)**.

Considering that AMTB may have potential off-target effects (Lashinger et al., 2008), we transfected HBECs with siRNA and Double Nickase plasmid to specifically knockdown and knockout TRPM8 in HBECs, respectively, to further explore the role of TRPM8. Treatment of HBECs with TRPM8 siRNA at two concentrations (50 and 100 nM) effectively reduced the basal TRPM8 expression (52 and 52% of the basal level, respectively) (Figure 2A). Additionally, treatment of HBECs with Double Nickase plasmid effectively reduced the basal TRPM8 expression at protein level and abolished its expression at mRNA levels (Figure 2C). Consistent with the findings from pretreatment with AMTB, the increased IL-8 production induced by Non-M-CSE or M-CSE was significantly reduced to similar levels by TRPM8 knockdown (Non-M-CSE vs. M-CSE, 430 vs. 494% of the basal level) (Figure 2B) and TRPM8 knockout (Non-M-CSE vs. M-CSE, 243 vs. 386% of the basal level) (Figure 2D). By contrast, transfection of scramble siRNA (Figure 2B) or control plasmid (Figure 2D) failed to alter the increased IL-8 production induced by either CSE. Moreover, perturbating TRPM8 gene by these two interventions did not affect the basal expression of IL-8 (Figures 2B,D).

### Roles of ROS and TRPM8 in the increased intracellular Ca^2+^ level induced by M-CSE or Non-M-CSE in HBECs

**Figure 3:**
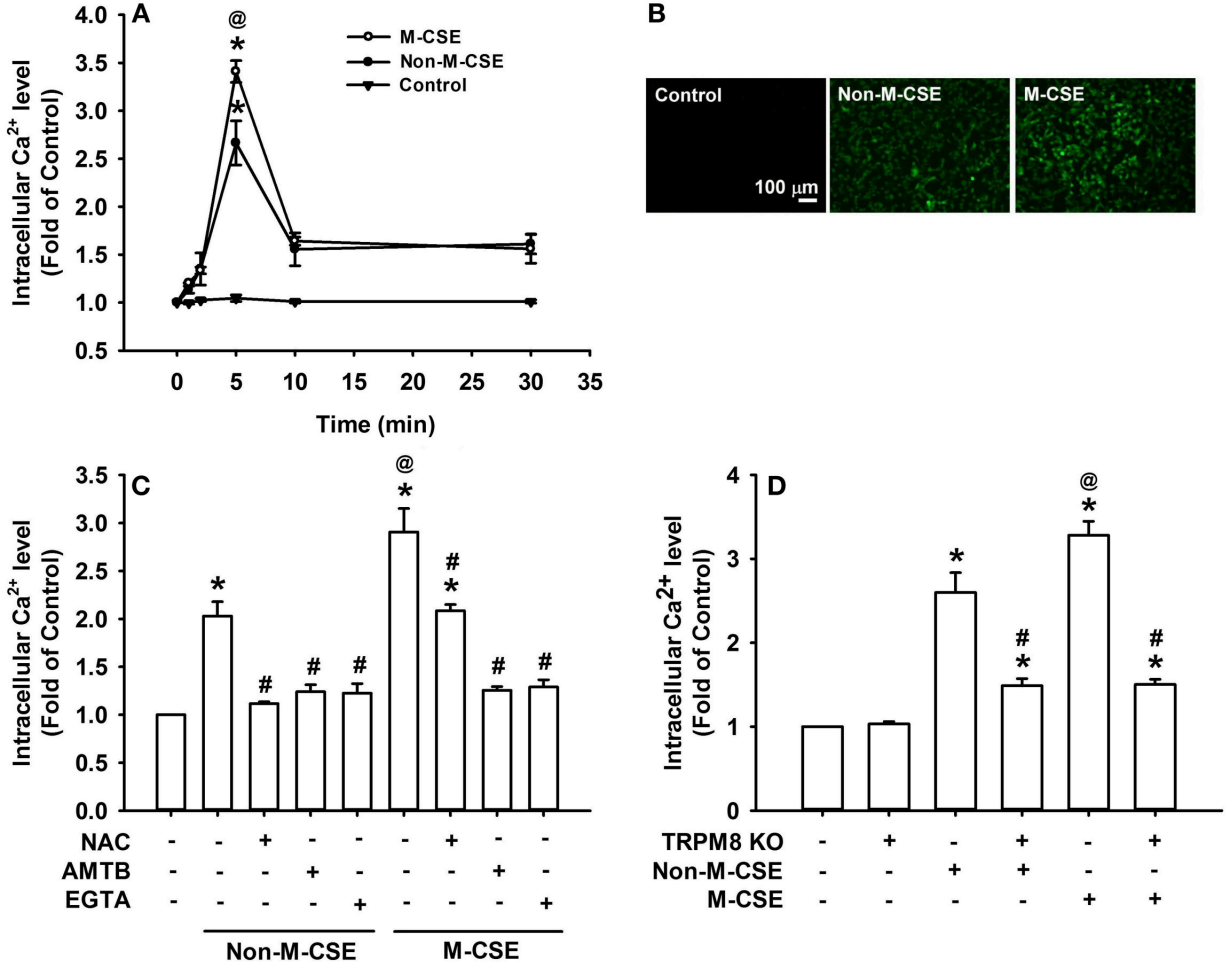
**Roles of ROS and TRPM8 in the increased intracellular Ca^2+^ level induced by M-CSE and Non-M-CSE in HBECs**. Intracellular Ca^2+^ levels were measured by Fluo-8 fluorescent probe assay. **(A)** Cells were exposed to medium alone (control), 1% Non-M-CSE, or 1% M-CSE for 1, 2, 5, 10, and 30 min. **(B)** Representative images of fluorescence-positive cells at 5 min after exposure. **(C)** Cells were pretreated with N-acetyl-cysteine (NAC), AMTB, or ethylene glycol tetraacetic acid (EGTA) for 1 h and then exposed to medium alone, 1% Non-M-CSE, or 1% M-CSE for 5 min. **(D)** Cells were transfected with control plasmid or TRPM8 Double Nickase plasmid (KO) and then exposed to medium alone, 1% Non-M-CSE, or 1% M-CSE for 5 min. Data from each group are means ± SEM from four independent experiments. ^*^p < 0.05 vs. the medium group **(A,C,D)**; ^@^*p* < 0.05 vs. the Non-M-CSE group without pretreatment **(A,C,D)**; #*p* < 0.05 vs. the same type of CSE group without pretreatment **(C,D)**.

Compared with the control, exposure of HBECs to Non-M-CSE or M-CSE increased the intracellular Ca^2+^ level, which reached its peak at 5 min after exposure (Figures 3A,B). Comparisons of intracellular Ca^2+^ responses to Non-M-CSE (260% of the basal level) and M-CSE (340% of the basal level) revealed the more remarkable effect of M-CSE at the concentration tested in HBECs (Figure 3A). Further analysis showed that the increased intracellular Ca^2+^ level induced by Non-M-CSE or M-CSE was totally inhibited by AMTB (Non-M-CSE vs. M-CSE, 124 vs. 126% of the basal level) or EGTA (Non-M-CSE vs. M-CSE, 123 vs. 129% of the basal level) (Figure 3C). Interestingly, NAC totally inhibited the increased intracellular Ca^2+^ level in Non-M-CSE-exposed cells (112% of the basal level), but only partially reduced the response in M-CSE-exposed cells (209% of the basal level) (Figure 3C). Consistent with the findings from drug pretreatment, TRPM8 knockout also significantly reduced the increased intracellular Ca^2+^ level induced by Non-M-CSE or M-CSE (Non-M-CSE vs. M-CSE, 145 vs. 146% of the basal level) (Figure 3D).

### Role of TRPM8 in the increased extracellular and intracellular ROS induced by M-CSE or Non-M-CSE in HBECs

**Figure 4:**
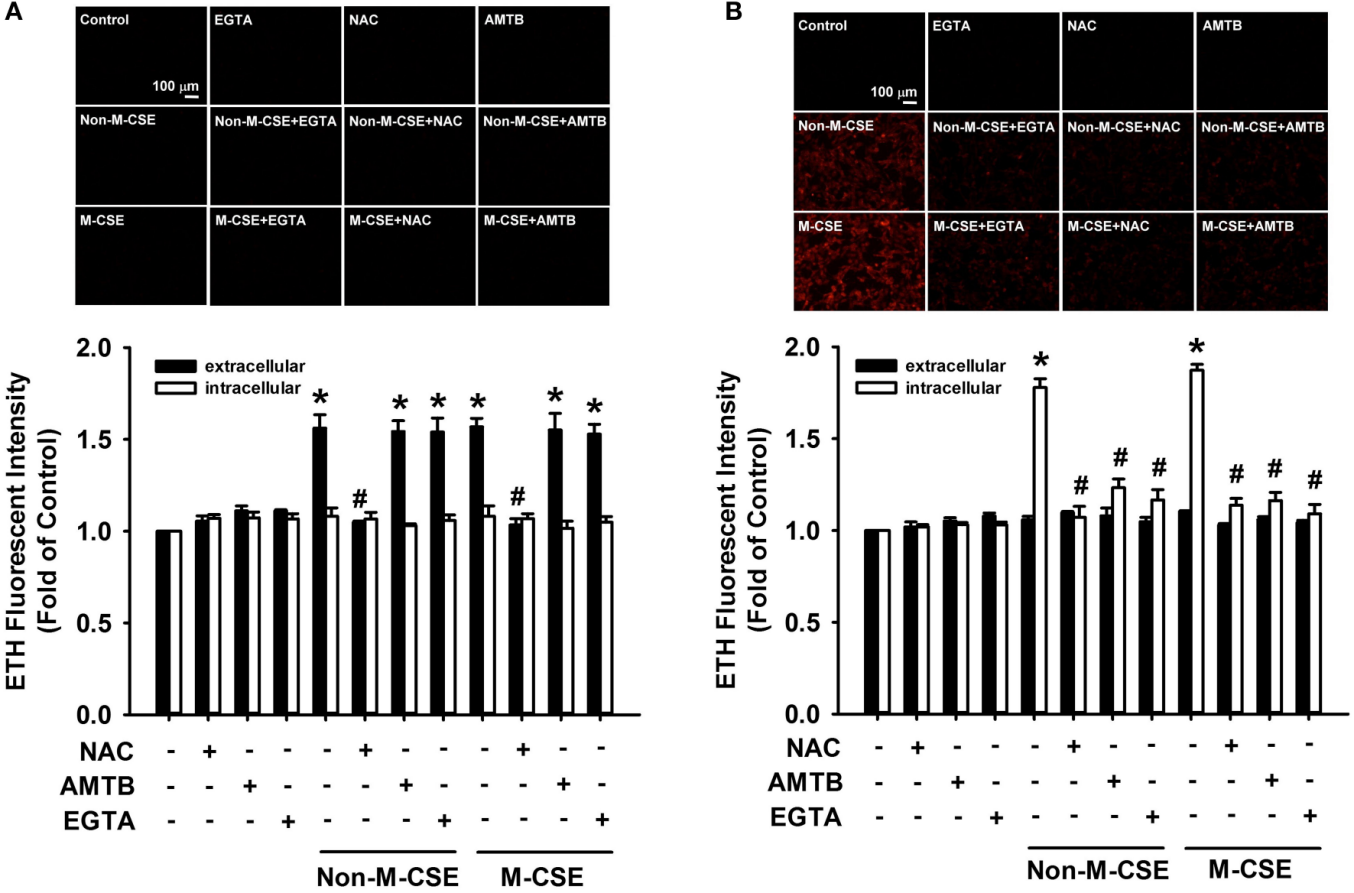
**Role of TRPM8 in the increased extracellular and intracellular ROS levels induced by M-CSE and Non-M-CSE in HBECs**. Cells were pretreated with NAC, AMTB, or EGTA for 1 h and then exposed to medium alone, 1% Non-M-CSE, or 1% M-CSE for 5 **(A)** and 30 min **(B)**. The ROS levels were assessed by the membrane-permeable probe hydroethidine, which was converted to red fluorescent ethidium (ETH) by ROS. The medium and cells were separately collected to measure the extracellular and intracellular levels of ROS, respectively. Data from each group are means ± SEM from four independent experiments. ^*^p < 0.05 vs. the medium group; #*p* < 0.05 vs. the same type of CSE group without pretreatment.

At 5 min after exposure to Non-M-CSE or M-CSE, the extracellular ROS level significantly increased in the medium containing HBECs (Non-M-CSE vs. M-CSE, 156 vs. 157% of the basal level), but the intracellular ROS level remained unchanged (Figure 4A). The increase in the extracellular ROS level was unaffected by pretreatment with AMTB or EGTA but was prevented by pretreatment with NAC (100% of the basal level) (Figure 4A). By contrast, at 30 min after exposure to Non-M-CSE or M-CSE, the intracellular ROS level significantly increased (Non-M-CSE vs. M-CSE, 178 vs. 187% of the basal level), while the extracellular ROS level returned to the baseline level (Figure 4B). This increase in intracellular ROS level was totally prevented by pretreatment with NAC (102% of the basal level), AMTB (113% of the basal level), or EGTA (106% of the basal level) (Figure 4B). The increases in extracellular and intracellular ROS levels induced by Non-M-CSE were similar to those induced by M-CSE (Figure 4).

### Roles of ROS and TRPM8 in the activation of the associated signaling pathway induced by M-CSE or Non-M-CSE in HBECs

**Figure 5:**
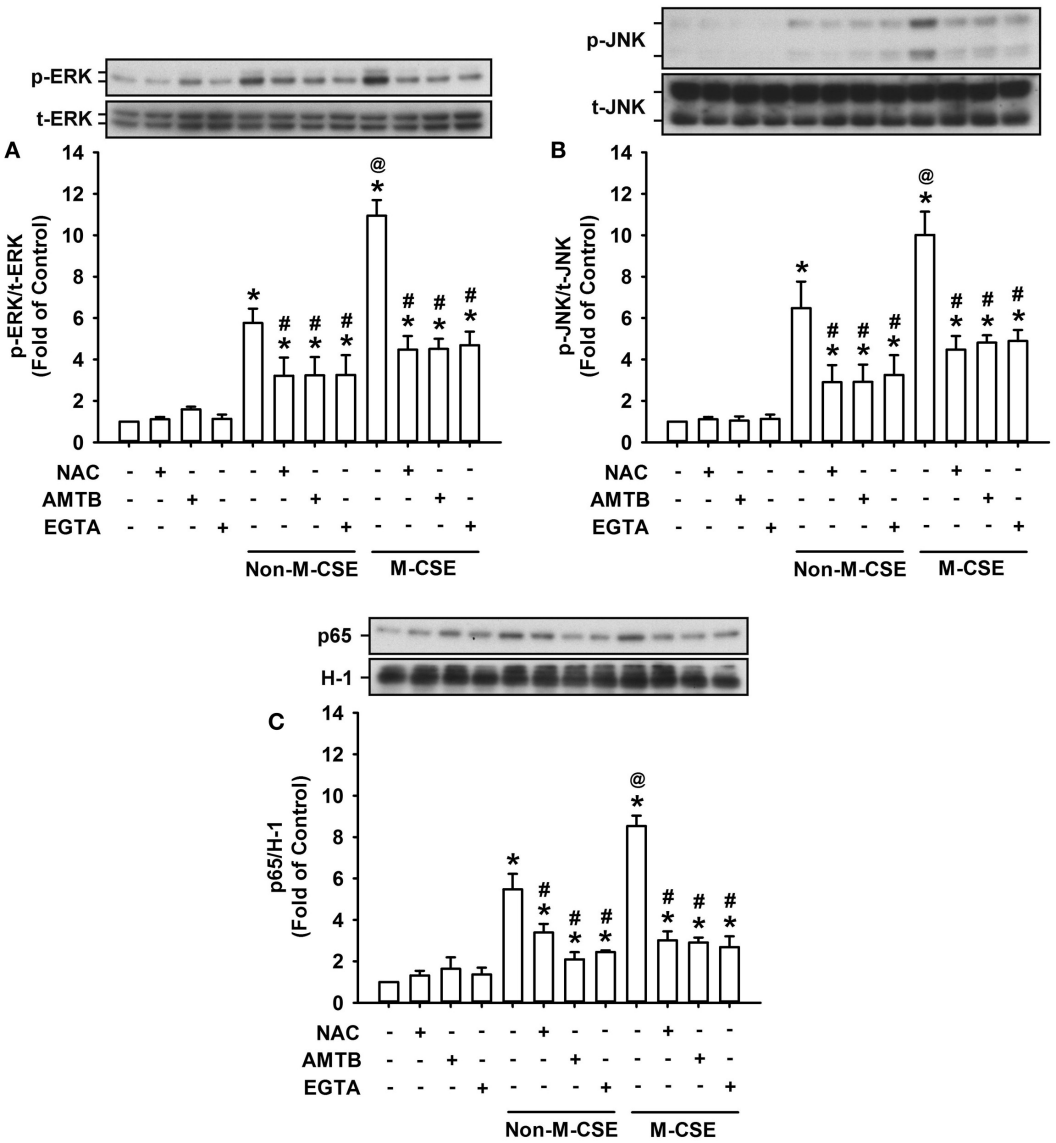
**Role of ROS and TRPM8 in the activation of the associated signaling pathway by M-CSE and Non-M-CSE in HBECs**. Cells were pretreated with NAC, AMTB, or EGTA for 1 h and then exposed to medium alone, 1% Non-M-CSE, or 1% M-CSE for 6 **(A,B)** and 12 h **(C)**, respectively. The activation of the signaling pathway is reflected by increases in phosphorylation of extracellular signal-regulated kinase (ERK) and c-Jun N-terminal kinase (JNK) in cell lysates and upregulation in the expression of p65 (a subunit of NF-κB) in nuclear extracts. Protein levels were analyzed by Western blot. p-, t-, and H-1 represent phospho-, total-, and histone H1 proteins, respectively. Data from each group are means ± SEM from four independent experiments. ^*^p < 0.05 vs. the medium group; ^@^*p* < 0.05 vs. the Non-M-CSE group without pretreatment; #*p* < 0.05 vs. the same type of CSE group without pretreatment.

The activation of ERK, JNK, and NF-κB is known to be a signaling pathway essential to the induction of IL-8 by CSE in HBECs (Mossman et al., 2006; Tang et al., 2011; Liu et al., 2014; Wu et al., 2014). Exposure of HBECs to Non-M-CSE or M-CSE resulted in increased phosphorylated ERK (Non-M-CSE vs. M-CSE, 576 vs. 1,094% of the basal level) (Figure 5A) and phosphorylated JNK (Non-M-CSE vs. M-CSE, 648 vs. 1,002% of the basal level) (Figure 5B) in the cytosol and the p65 subunit of NF-κB in the nucleus (Non-M-CSE vs. M-CSE, 548 vs. 854% of the basal level) (Figure 5C). Comparisons of the responses of these signaling regulators to Non-M-CSE and M-CSE revealed the more remarkable effect of M-CSE at the concentration tested in HBECs (Figure 5). Such CSE-induced activation of the MAPKs/NF-κB signaling to either type of CSE was significantly attenuated to a similar level by pretreatment with NAC (Non-M-CSE vs. M-CSE, 290 vs. 403% of the basal level), AMTB (Non-M-CSE vs. M-CSE, 203 vs. 284% of the basal level), or EGTA (Non-M-CSE vs. M-CSE, 289 vs. 415% of the basal level) (Figure 5). No differences in the residual responses to Non-M-CSE and M-CSE were detected (Figure 5).

### Responses of intracellular Ca^2+^ and IL-8 to menthol alone or in combination with Non-M-CSE in HBECs

**Figure 6:**
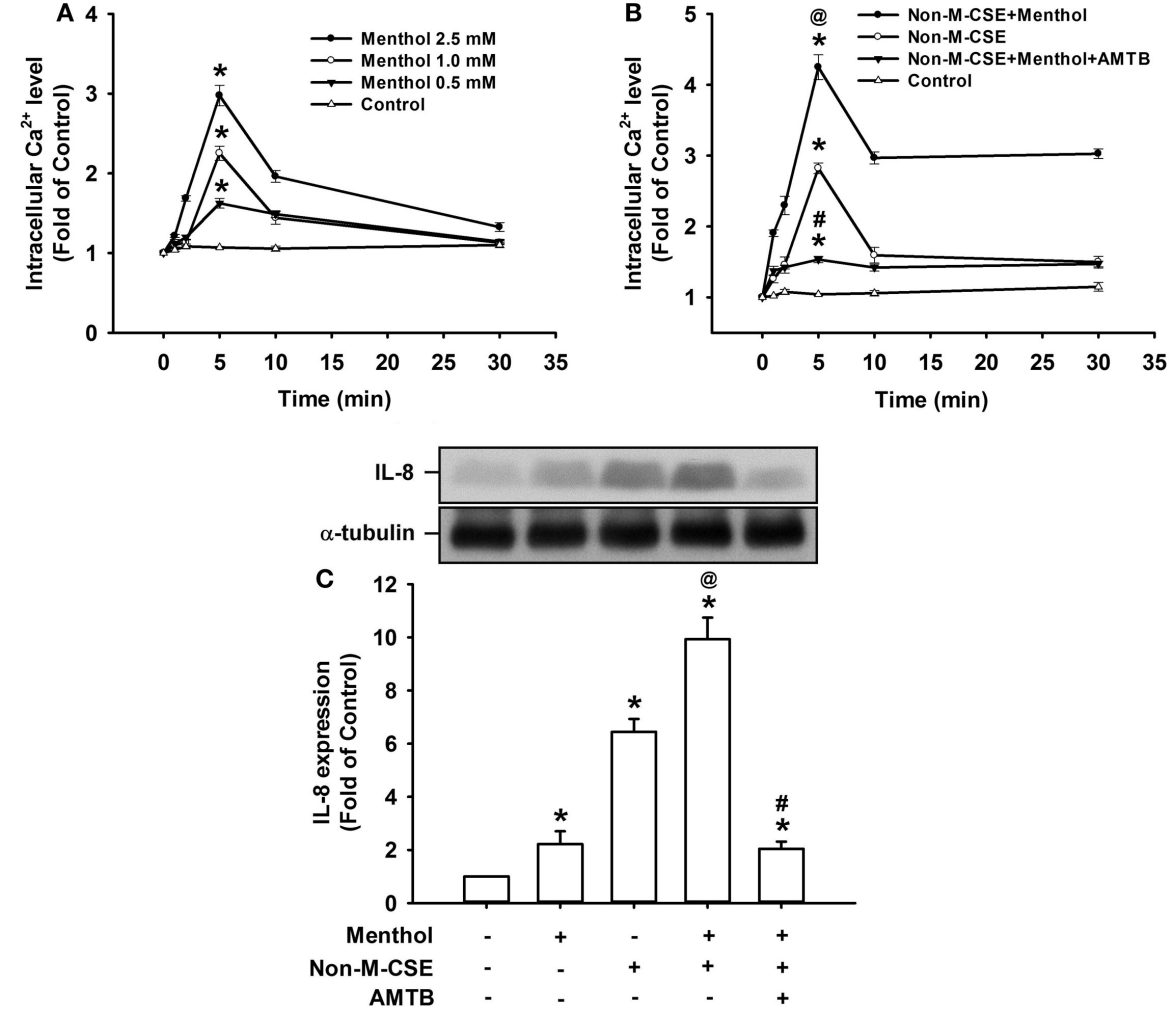
**Responses of intracellular Ca^2+^ and IL-8 to menthol alone or in combination with Non-M-CSE in HBECs**. Intracellular Ca^2+^ levels were measured by Fluo-8 fluorescent probe assay at 1, 2, 5, 10, and 30 min after exposure. IL-8 levels were analyzed by Western blot at 24 h after exposure. Pretreatment with AMTB was performed 1 h prior to exposure. Cells were exposed to medium alone (control) or menthol (0.5–2.5 mM) **(A)**, medium alone, 1% Non-M-CSE, or a combination of Non-M-CSE and menthol (2.5 mM) **(B)**, or medium alone, menthol (2.5 mM), 1% Non-M-CSE, or a combination of Non-M-CSE and menthol **(C)**. Data in each group are means ± SEM from four independent experiments. ^*^p < 0.05 vs. the medium group; ^@^*p* < 0.05 vs. the Non-M-CSE group without pretreatment; #*p* < 0.05 vs. the combination group without pretreatment.

Compared with the control, exposure of HBECs to menthol alone caused a concentration-dependent increase in intracellular Ca^2+^ level, which reached its peak at 5 min after exposure (298% of the basal level for 2.5 mM concentration) (Figure 6A). The increase in intracellular Ca^2+^ level at 5 min after exposure (425% of the basal level) (Figure 6B) or induction of IL-8 at 24 h after exposure (993% of the basal level) (Figure 6C) induced by a combination of Non-M-CSE and menthol (2.5 mM) was greater than by Non-M-CSE alone (intracellular Ca^2+^, 282%; IL-8, 645% of the basal level). The Ca^2+^ and IL-8 responses were largely suppressed by pretreatment with AMTB (Figures 6B,C).

### Responses of intracellular Ca^2+^ and IL-8 to M-CSE or Non-M-CSE in HEK293 cells transfected with TRPM8

**Figure 7:**
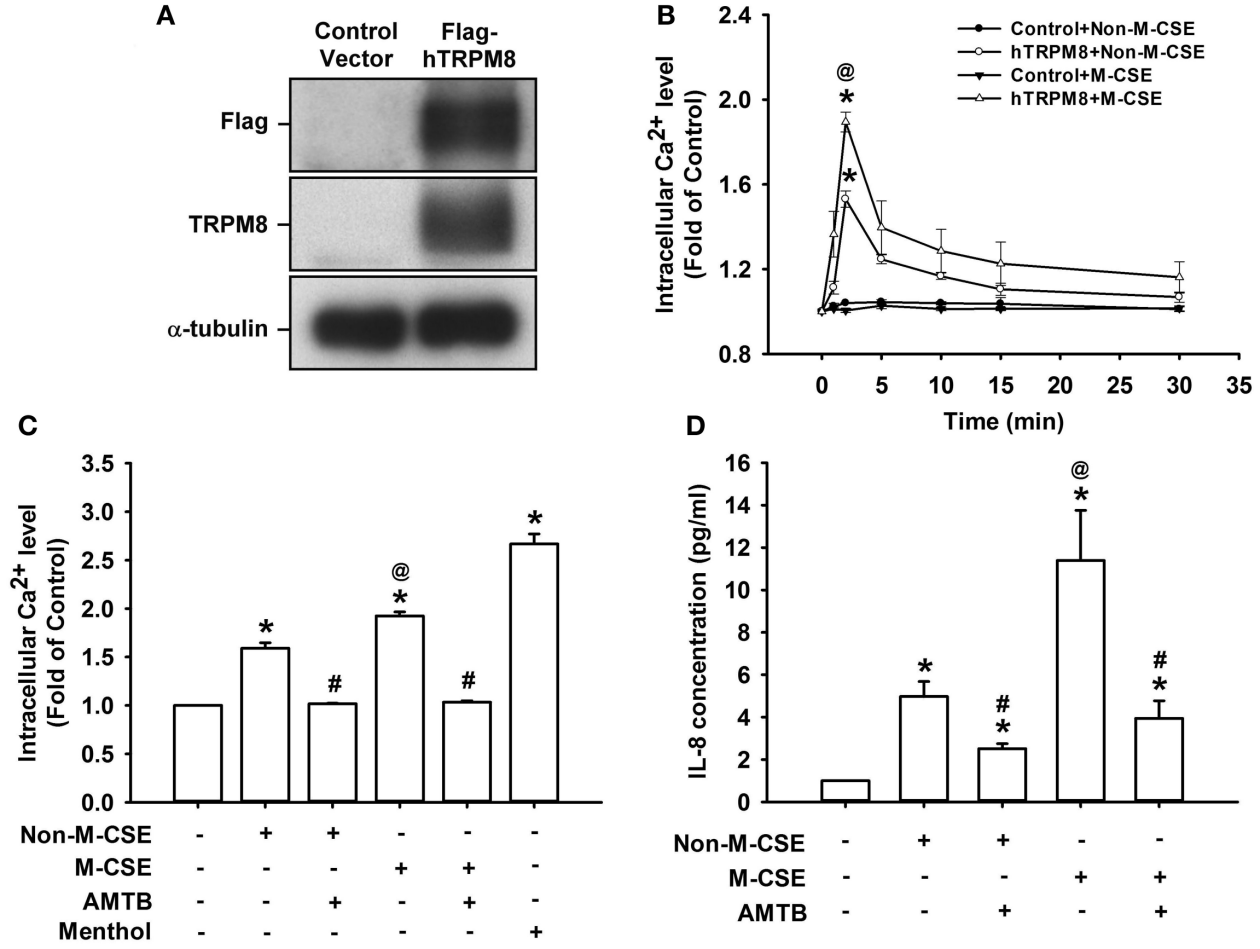
**Responses of intracellular Ca^2+^ and IL-8 to M-CSE and Non-M-CSE in HEK293 cells transfected with human TRPM8 (hTRPM8). (A)** Cells were transfected with control (pCMV6) or Flag-tagged hTRPM8 vector for 24 h. Anti-Flag and anti-TRPM8 antibodies were used to perform Western blot to confirm successful transfection. **(B)** Control cells or hTRPM8-expressing cells were exposed to 1% Non-M-CSE or M-CSE for 1, 2, 5, 10, and 30 min. Intracellular Ca^2+^ levels were measured by Fluo-8 fluorescent probe assay. The hTRPM8-expressing cells were exposed to medium alone, 1% Non-M-CSE, 1% M-CSE, or menthol for 2 min (**C)** and 24 h **(D)** with or without pretreatment with AMTB. IL-8 levels were analyzed by ELISA. Data from each group are means ± SEM from four independent experiments. ^*^p < 0.05 vs. the control vector group **(B)** or medium group **(C,D)**; ^@^*p* < 0.05 vs. the Non-M-CSE group without pretreatment **(B–D)**; #*p* < 0.05 vs. the same type of CSE group without pretreatment **(C,D)**.

Considering that HBECs express other CSE-sensitive transient receptor potential (TRP) channels, such as TRP ankyrin 1 (TRPA1) (Nassini et al., 2012; Lin et al., 2015), we transfected HEK293 cells with hTRPM8 to further explore the importance of TRPM8. Cells transfected with hTRPM8 vector successfully expressed TRPM8, contrary to the failure of cells transfected with the control vector (Figure 7A). Similar to the results using HBECs, exposure of hTRPM8-expressing cells to Non-M-CSE or M-CSE caused an increase in intracellular Ca^2+^ level, with more remarkable response shown by cells exposed to M-CSE (Non-M-CSE vs. M-CSE, 153 vs. 189% of the basal level) (Figure 7B). By contrast, exposure of control cells to Non-M-CSE or M-CSE did not alter the intracellular Ca^2+^ level (Figure 7B). Exposure of hTRPM8-expressing cells to menthol alone also increased intracellular Ca^2+^ level, and these cells could serve as the positive control (Figure 7C). Similarly, exposure of hTRPM8-expressing cells to Non-M-CSE or M-CSE caused an increase in IL-8 production, and the response was also greater in cells exposed to M-CSE (Non-M-CSE vs. M-CSE, 497 vs. 1,139% of the basal level) (Figure 7D). Further analysis revealed that pretreatment with AMTB totally inhibited the increased intracellular Ca^2+^ level (Non-M-CSE vs. M-CSE, 102 vs. 103% of the basal level) (Figure 7C) and induction of IL-8 by Non-M-CSE or M-CSE (Non-M-CSE vs. M-CSE, 251 vs. 394% of the basal level) (Figure 7D).

### Oxidative stress-related events induced by M-CSE or Non-M-CSE in HBECs

**Figure 8:**
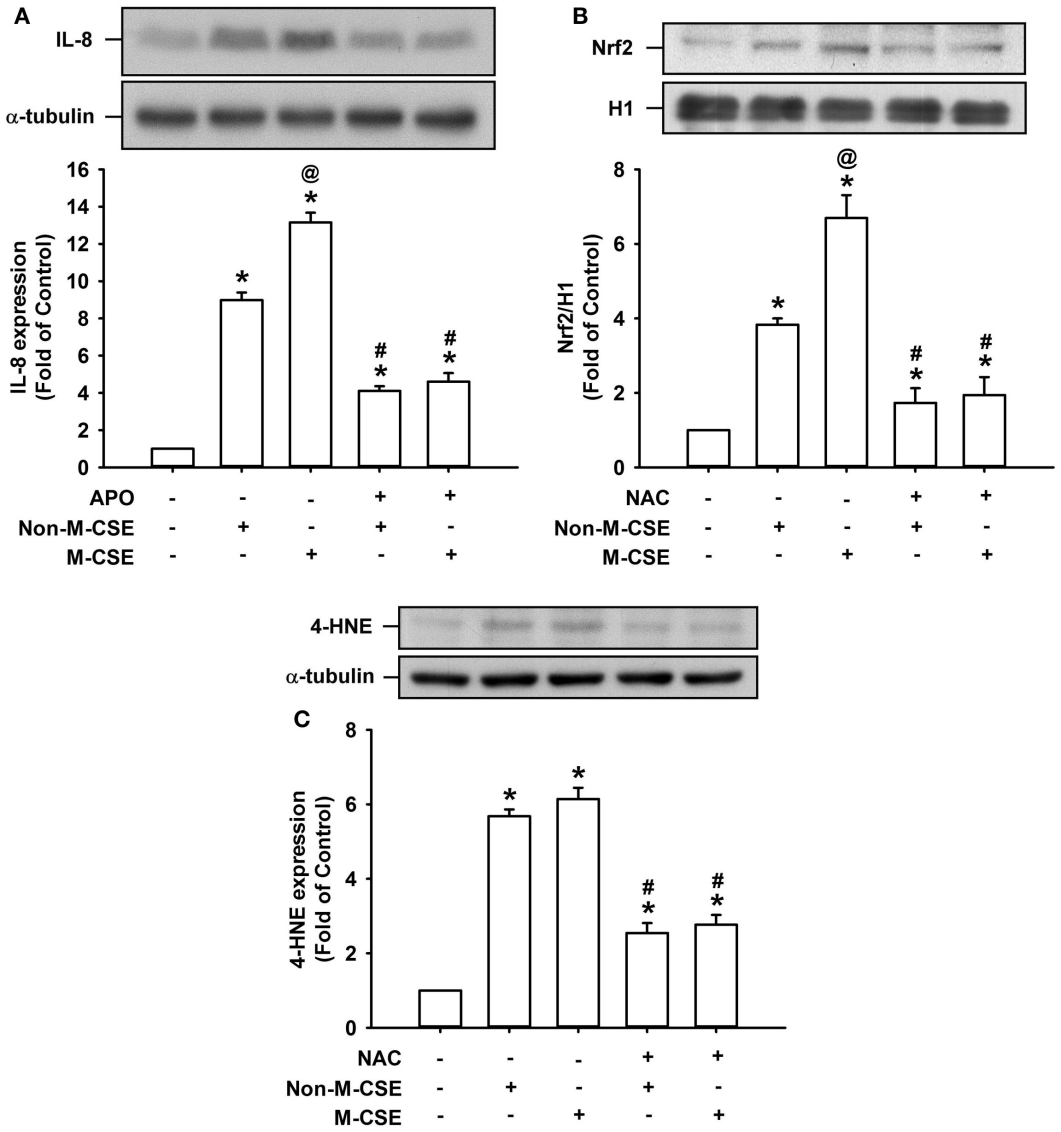
**Oxidative stress-related events induced by M-CSE and Non-M-CSE in HBECs. (A)** Cells were pretreated with apocynin (APO, a NADPH oxidase inhibitor; 150 μM) for 1 h and then exposed to medium alone, 1% Non-M-CSE, or M-CSE for 24 h. **(B,C)** Cells were pretreated with N-acetyl-cysteine (NAC) for 1 h and then exposed to medium alone, 1% Non-M-CSE, or M-CSE for 2 h **(B)** or for 24 h **(C)**. Levels of IL-8 and 4-HNE were measured using cell lysates, and the level of Nrf2 was measured using nuclear extracts. Protein levels were analyzed by Western blot. Data from each group are means ± SEM from four independent experiments. ^*^p < 0.05 vs. the medium group **(A–C)**; ^@^*p* < 0.05 vs. the Non-M-CSE group without pretreatment **(A,B)**; #*p* < 0.05 vs. the same type of CSE group without pretreatment **(A–C)**.

We further studied certain oxidative stress-related events induced by both types of CSE. Comparisons of the IL-8 responses to Non-M-CSE (899% of the basal level) and M-CSE (1315% of the basal level) revealed that M-CSE exerted a greater effect (Figure 8A). The increased IL-8 production induced by Non-M-CSE or M-CSE was significantly reduced to a similar level by a NAPDH oxidase inhibitor, apocynin (Non-M-CSE vs. M-CSE, 410 vs. 460% of the basal level) (Figure 8A). Comparisons of the responses of Nrf2, a redox sensor, to Non-M-CSE (383% of the basal level) and M-CSE (670% of the basal level) revealed that M-CSE exerted a greater effect (Figure 8B). The increased Nrf2 level induced by Non-M-CSE or M-CSE was significantly reduced to a similar level by NAC (Non-M-CSE vs. M-CSE, 173 vs. 194% of the basal level) (Figure 8B). Comparisons of the responses of 4-HNE, an oxidative stress biomarker, to Non-M-CSE (568% of the basal level) and M-CSE (614% of the basal level) revealed that both types of CSE had a similar effect (Figure 8C). The increased 4-HNE expression induced by Non-M-CSE or M-CSE was significantly reduced by NAC (Non-M-CSE vs. M-CSE, 254% vs. 277% of the basal level) (Figure 8C).

## Discussion

In this study, exposure of HBECs to M-CSE or Non-M-CSE sequentially induced several events, such as increases in extracellular ROS, intracellular Ca^2+^ level via ion influx, and intracellular ROS. These events were followed by the activation of the MAPK/NF-κB signaling and, ultimately, the induction of IL-8. These CSE-induced events exhibited upstream and downstream relationships in our previous studies (Tang et al., 2011; Liu et al., 2014; Wu et al., 2014; Lin et al., 2015). We used this *in vitro* model to compare the inflammatory effects of M-CSE and Non-M-CSE and delineate the mechanisms underlying the more negative impact of M-CSE.

M-CSE induced greater IL-8 induction in HBECs compared with Non-M-CSE at the same concentration (Figure 1D). The augmented IL-8 response induced by M-CSE may have resulted from a more rigorous activation of the MAPK/NF-κB signaling (Figure 5), because this inflammatory signaling is vital for IL-8 induction in HBECs (Liu et al., 2014; Lin et al., 2015). All responses to Non-M-CSE or M-CSE were reduced to similar levels by antagonizing TRPM8 with AMTB or removing extracellular Ca^2+^ with EGTA. No differences were noted in the residual responses to Non-M-CSE and M-CSE, suggesting the important role of TRPM8 in these responses. Thus, we speculated that the part of the responses augmented by M-CSE may have been caused by the additional activation of TRPM8 by menthol in M-CSE. We also postulated that ROS derived from Non-M-CSE or M-CSE could induce these responses via activation of TRPM8. This hypothesis is strongly supported by the current results, which show that, similar to AMTB or EGTA, scavenging ROS with NAC could suppress these responses (Figures 1D, 5). Moreover, this concept is in good agreement with the findings that TRPM8 in the uroepithelium could be activated by direct exposure to H_2_O_2_ (Nocchi et al., 2014). Of note, AMTB has been considered to have potential off-target effects (Lashinger et al., 2008). In addition, CSE may activate other TRP channels, such as TRPA1 in HBECs (Nassini et al., 2012; Lin et al., 2015). The essential role of TRPM8 in IL-8 induction by Non-M-CSE or M-CSE and the difference in IL-8 responses between these two CSE types were confirmed by our findings in HBECs with TRPM8 knockdown (Figure 2B) and TRPM8 knockout (Figure 2D), as well as in HEK293 cells transfected with hTRPM8 (Figure 7D).

We then investigated the possible mechanisms on how TRPM8 was activated by Non-M-CSE or M-CSE in HBECs. Non-M-CSE or M-CSE increased extracellular ROS as early as 5 min after exposure (Figure 4A). However, the level of intracellular ROS remained unaltered at this time point. These results are consistent with our previous findings (Lin et al., 2015). The extracellular ROS response to Non-M-CSE was similar to that to M-CSE, and both responses were eliminated by NAC, indicating the equivalent oxidant potency of these two types of CSE. This phenomenon also suggests that the increased extracellular ROS was generated by CSE *per se* but not by the menthol in M-CSE. Although the extracellular ROS responses to both types of CSE were similar, Non-M-CSE or M-CSE subsequently increased intracellular Ca^2+^ within 5 min after exposure, and the response to M-CSE was greater than that to Non-M-CSE (Figure 3C). This increase in intracellular Ca^2+^ may have resulted from the activation of TRPM8, because the response was inhibited by AMTB, EGTA (Figure 3C), or TRPM8 knockout (Figure 3D). NAC totally inhibited the intracellular Ca^2+^ response to Non-M-CSE but only partially suppressed this response to M-CSE. Thus, ROS is apparently the common stimulus for both types of CSE to activate TRPM8, and that the menthol existing in M-CSE may serve as an additional stimulus for this activation, resulting in greater intracellular Ca^2+^ response. Similar ROS-related regulation of other types of TRP channels has been reported elsewhere (Song et al., 2011; Lin et al., 2015). Thus, the greater intracellular Ca^2+^ response to M-CSE may contribute to the more rigorous activation of MAPK/NF-κB signaling observed in our study, because intracellular Ca^2+^ can activate this signaling pathway in HBECs (Carmona et al., 2010; Lin et al., 2015). Moreover, direct exposure to menthol alone increased intracellular Ca^2+^ level and IL-8 induction, and combined menthol and Non-M-CSE induced greater responses compared with Non-M-CSE alone (Figure 6), suggesting that Non-M-CSE has the capacity to induce greater responses if menthol is present in CSE. It was not our intension to correlate the effects of menthol alone to M-CSE because we do not know the concentration of menthol in the M-CSE during preparation. These observations support our hypothesis that menthol in M-CSE may serve as an additional stimulus to activate TRPM8, although the concentration of menthol given directly was higher than that in M-CSE. The induction of inflammatory responses via TRPM8 by direct exposure of HBECs to menthol alone has been previously reported (Sabnis et al., 2008a,b; Li et al., 2011).

Following the increase in intracellular Ca^2+^, Non-M-CSE or M-CSE increased intracellular ROS within 30 min after exposure. This response was also totally prevented by AMTB or EGTA (Figure 4B), suggesting the importance of TRPM8 and intracellular Ca^2+^. The increased intracellular ROS has been reported to be resulted from the activation of NADPH oxidase (Tang et al., 2011; Liu et al., 2014; Lin et al., 2015) and was presumed to be triggered by the TRPM8-mediated increase in intracellular Ca^2+^. Indeed, the Ca^2+^ signaling mediated by various Ca^2+^ channels can regulate the activity of NADPH oxidase (Jiang et al., 2011). The menthol-induced, TRPM8-mediated increase in intracellular Ca^2+^ can promote the elevation of intracellular ROS in fibroblasts (Zhu et al., 2014). The absence of difference between the responses of intracellular ROS to M-CSE and Non-M-CSE (Figure 4B) may be due to the ceiling effect of the production of intracellular ROS. Thus, lung epithelial TRPM8 may provide an important link between the initial increase in extracellular ROS and subsequent increase in intracellular ROS via the Ca^2+^ signaling in our model. The increase in intracellular ROS induced by Non-M-CSE and M-CSE may have also contributed to the activation of MAPK/NF-κB signaling observed in our study, because intracellular ROS have been reported to have this function (Tang et al., 2011; Wu et al., 2014; Lin et al., 2015).

We further studied certain oxidative stress-related events induced by both types of CSE. Similar to the responses of IL-8, M-CSE exerted a greater effect on the expression of Nrf2 (a redox sensor), but not 4-HNE (an oxidative stress biomarker), as compared to Non-M-CSE (Figure 8). The increased IL-8 production induced by Non-M-CSE or M-CSE was reduced to a similar level by apocynin, suggesting the involvement of NAPDH oxidase in the IL-8 responses to these two types of CSE. The increased levels of Nrf2 and 4-HNE induced by Non-M-CSE or M-CSE were also reduced to a similar level by NAC, indicating the equivalent oxidant potency of these two types of CSE.

Menthol can counteract airway irritation in animals (Willis et al., 2011; Ha et al., 2015; Liu et al., 2015) and human (Millqvist et al., 2013) and produce cooling sensation (Lawrence et al., 2011). These smoothing properties support the hypothesis that menthol cigarette smoking may result in larger puffs, deeper inhalation, or longer retention time in the lung, thereby producing more adverse effects compared with non-menthol cigarette smoking (Hoffman, 2011; Lawrence et al., 2011; Besaratinia and Tommasi, 2015). However, results from studies on the smoking topography and blood biomarkers of smoke exposure in animals (Gaworski et al., 1997; Ha et al., 2015) and smokers (Heck, 2009, 2010; Caraballo et al., 2011; Hoffman, 2011; Lawrence et al., 2011) are inconclusive to support this hypothesis. Results from a clinical investigation have suggested that menthol cigarette smokers may exhibit more severe lung inflammation than non-menthol cigarette smokers in patients with COPD (Park et al., 2015). Our findings that, in addition to ROS, the menthol in M-CSE may have extra inflammatory effects on HBECs provide an alternative evidence to support the concept on the more negative impact of menthol cigarette smoking on the lungs (Hoffman, 2011).

The concentration of menthol in the cell medium with M-CSE exposure is not known in this study. However, the differences in various responses to Non-M-CSE and M-CSE can be attributed to the menthol in M-CSE, because these two types of CSE have equal potency of oxidant properties as demonstrated in this study. Additionally, we recently reported that Non-M-CSE may also activate lung epithelial TRPA1 and increase intracellular Ca^2+^ via influx, which ultimately promote IL-8 production (Lin et al., 2015). In this study, the increased intracellular Ca^2+^ response to Non-M-CSE or M-CSE was prevented by AMTB, suggesting that the inflammatory role of TRPA1 was downplayed. The exact mechanism for this masking effect remains unclear. TRPM8 may have structurally interacted with TRPA1, and activation of one type of TRP channel could have inhibited the function of the other type (Harrington et al., 2011). TRPA1 have been shown to structurally interact with TRPV1, another type of TRP channels (Staruschenko et al., 2010; Fischer et al., 2014). The other limitation of this study is that we did not use non-menthol and menthol cigarettes from the same brand. This is because, in reality, we could not found these two types of cigarettes from the same brand with similar characteristics, including the content of tar, content of nicotine, length, diameter, and amount of tobacco in each cigarette. We therefore decided to use these two types of cigarettes with very similar characteristics from two different brands.

In summary, we demonstrated that, compared with exposure to Non-M-CSE, exposure to M-CSE induced greater TRPM8-mediated responses, including increases in intracellular Ca^2+^, activation of ROS-sensitive MAPK/NF-κB signaling, and induction of IL-8 in HBECs. The augmented inflammatory effects of M-CSE may be due to a double-hit on lung epithelial TRPM8 by ROS generated from CSE and menthol in M-CSE.

## Author contributions

AL, ML, HK, and DP conducted the studies and analyzed and interpreted the data. ML and AL wrote the paper. TL and YK led the project, interpreted the data, and finished the manuscript.

### Conflict of interest statement

The authors declare that the research was conducted in the absence of any commercial or financial relationships that could be construed as a potential conflict of interest. The reviewer SV and handling Editor declared their shared affiliation, and the handling Editor states that the process nevertheless met the standards of a fair and objective review.
